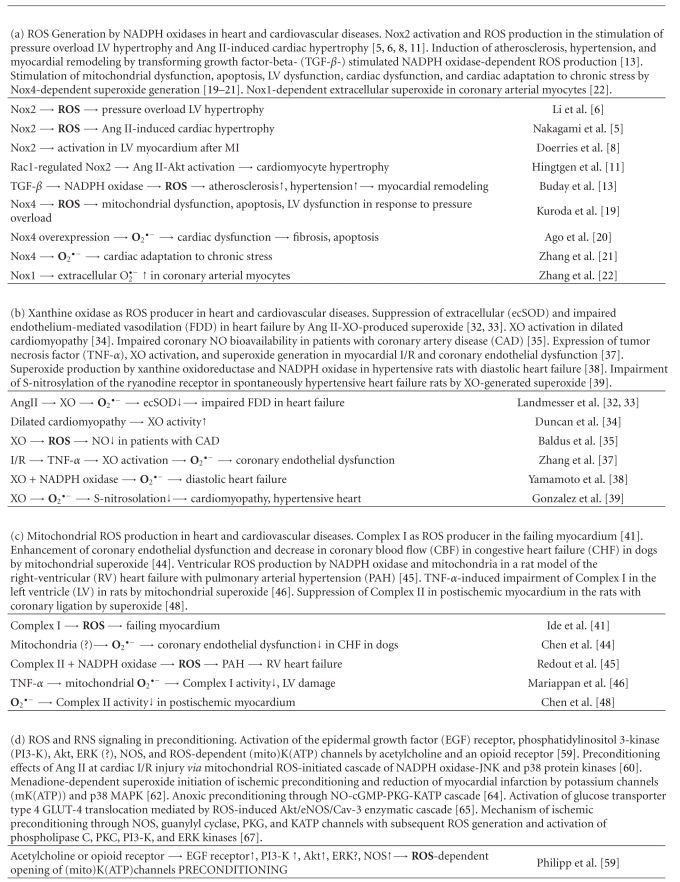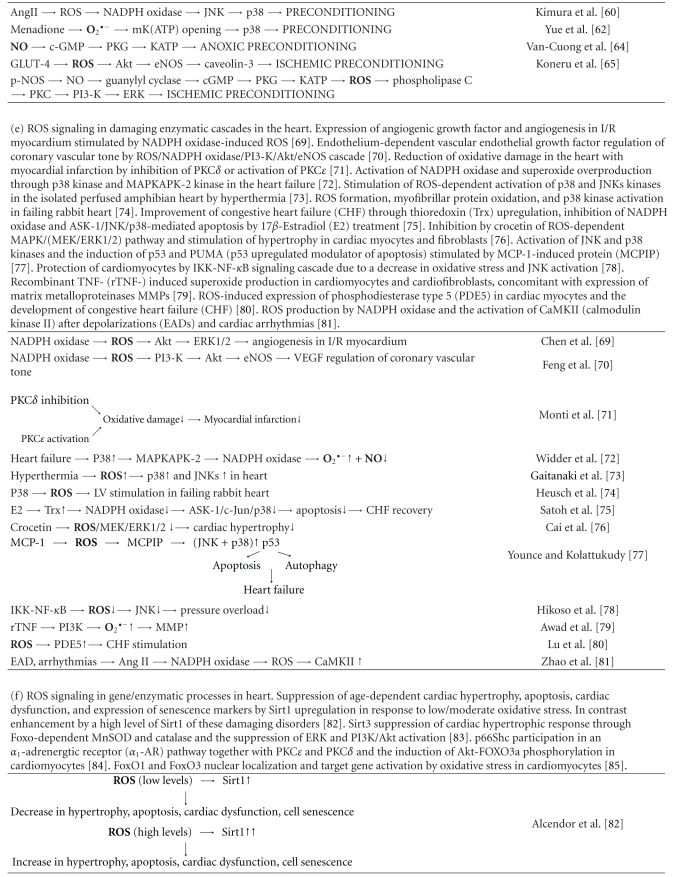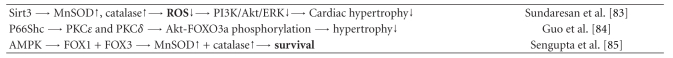# ROS and RNS Signaling in Heart Disorders: Could Antioxidant Treatment Be Successful?

**DOI:** 10.1155/2011/293769

**Published:** 2011-09-08

**Authors:** Igor Afanas'ev

**Affiliations:** Vitamin Research Institute, Moscow, Russia

## Abstract

There is not too much success in the antioxidant treatment of heart deceases in humans. However a new approach is now developed that suggests that depending on their structures and concentrations antioxidants can exhibit much more complicated functions in many pathological disorders. It is now well established that physiological free radicals superoxide and nitric oxide together with their derivatives hydrogen peroxide and peroxynitrite (all are named reactive oxygen species (ROS) and reactive nitrogen species (RNS)) play a more important role in heart diseases through their signaling functions. Correspondingly this work is dedicated to the consideration of damaging signaling by ROS and RNS in various heart and vascular disorders: heart failure (congestive heart failure or CHF), left ventricular hypertrophy (LVH), coronary heart disease, cardiac arrhythmias, and so forth. It will be demonstrated that ROS overproduction (oxidative stress) is a main origin of the transformation of normal physiological signaling processes into the damaging ones. Furthermore the favorable effects of low/moderate oxidative stress through preconditioning mechanisms in ischemia/reperfusion will be considered. And in the last part we will discuss the possibility of efficient application of antioxidants and enzyme/gene inhibitors for the regulation of damaging ROS signaling in heart disorders.

## 1. Introduction

Heart disease (cardiopathy) and cardiovascular diseases are a group of numerous pathological disorders such as heart failure (congestive heart failure or CHF), left ventricular hypertrophy (LVH), coronary heart disease, cardiac arrhythmias, and so forth, in which signaling processes of reactive oxygen and reactive nitrogen species (ROS and RNS) play an important (probably critical) role. Contemporary studies identified major sources of ROS and RNS productions: NADPH oxidases (Nox), xanthine oxidase, mitochondria, and nitric oxide synthases (NOS). As a rule, heart and cardiovascular diseases are characterized by ROS overproduction whereas the formation of major RNSs nitric oxide (a free radical) and peroxynitrite (diamagnetic molecule) can decrease or increase depending on the nature of heart injury. Free radicals are usually considered to be the damaging factors in various pathologies, but on the other hand ROS and RNS are important signaling species in many physiological and pathophysiological processes. For example the critical role of these species has been shown in preconditioning and other survival processes (see below). A major aim of this work is to consider the role of ROS and RNS signaling in various heart and cardiovascular diseases.

## 2. NADPH Oxidases as ROS Producers in Heart and Cardiovascular Diseases

NADPH oxidases generate superoxide by the one-electron reduction of dioxygen:

(1) $$\begin{array}{c} 2{\text{O}}_{2}+\text{NADPH}\to 2{{\text{O}}_{2}}^{•-}+{\text{NADP}}^{+}+{\text{H}}^{+} \end{array}$$

Family of NADPH oxidases (Nox) consists of several isoenzymes. In addition to phagocyte NADPH oxidase (Nox2), six homologs (Nox1, Nox3, Nox4, Nox5, Duox1, and Duox2) are now identified in nonphagocytic cells; however, their role in cardiovascular and heart diseases might be quite different. ROS generation by NADPH oxidases in heart diseases has previously been discussed [1–3]. It has been suggested that the Nox-dependent ROS signaling is an important factor responsible for the development of many pathological processes in heart.

### 2.1. Phagocyte NADPH Oxidase Nox2

Phagocyte NADPH oxidase Nox2 plays important role in heart injury. Bendall et al. [4] found that Nox2 (gp91^phox^) was an important factor of the development of Ang II-induced cardiac hypertrophy independently of the change in blood pressure in mice. Similar effect of NADPH oxidase-derived superoxide was demonstrated by Nakagami et al. [5] Li et al. [6] showed that ROS generation by phagocyte NADPH oxidase in cardiomyocytes induced the pressure overload LV hypertrophy. The elevated expression of NADPH oxidase and superoxide production was found in the carotid body from rabbits with chronic heart failure [7]. Similarly Doerries et al. [8] demonstrated the enhanced activity of NADPH oxidase subunit p47phox (Nox2) in the mouse left ventricular (LV) myocardium after myocardial infarction (MI). While Nox2 was required for the response to Ang II-induced left ventricular hypertrophy (LVH), another NADPH oxidase isoform Nox4 was apparently involved in the cardiac response to pressure overload in murine myocardium [9]. 

 It was found that Rac1 initiated hypertrophic response in the heart dependent on NADPH oxidase-generated ROS [10]. Hingtgen et al. [11] confirmed that superoxide production by a Rac1-regulated Nox2 initiated the Ang II-induced activation of protein kinase Akt in cardiomyocyte hypertrophy. Judkins et al. [12] showed that Nox2 was responsible for vascular ROS production, reduced NO bioavailability, and the early lesion development in aorta of the mice. Buday et al. [13] found that the elevated circulating transforming growth factor beta (TGF-*β*) induced NADPH oxidase activation and ROS overproduction that accelerated atherosclerosis, hypertension, and myocardial remodeling in apolipoprotein E-deficient (apoE(−/−) mice.

### 2.2. NADPH Oxidase Nox4

NADPH oxidase Nox4 is a NADPH oxidase isoenzyme which plays an important role in heart and vascular diseases. Martyn et al. [14] suggested that Nox4 connected to the protein p22^phox^  on internal membranes in epithelial cells. It was also found that in contrast to the other NADPH oxidase isoforms Nox4 produced mainly hydrogen peroxide and the very small amounts of superoxide. Cytosolic oxidase proteins or the GTPase Rac are not required for the activity of this enzyme. Serrander et al. [15] also found that Nox4 produced hydrogen peroxide but also generated superoxide intracellularly. 

 There is a lot of uncertainty in the studies of Nox4-dependent ROS production. Conclusion that Nox4 produces mainly hydrogen peroxide and not superoxide contradicts a majority of the other experimental data. It is possible that unreliable methods such as nitroblue tetrazolium (NBT) reduction were applied for superoxide detection in the above works [14–16]. On the other hand the use of the most specific and precise method of lucigenin chemiluminescence (CL) for superoxide detection gave different results [17]. For example, using lucigenin CL, Guzik et al. [18] found that Nox4 and Nox2 produced about 75% superoxide in coronary arteries from patients with coronary artery disease (CAD). 

 Nox4 is apparently located in different way in cardiac cells comparing to other NADPH oxidases. Thus Kuroda et al. [19] found that Nox4 was localized in mitochondria and was a major source of superoxide production in cardiac myocytes. Upregulation of Nox4 increased mitochondrial superoxide in response to pressure overload (PO). These authors also showed that Nox4 induced mitochondrial dysfunction, apoptosis in cardiac myocytes, and LV dysfunction in response to PO. Ago et al. [20] demonstrated that Nox4 was upregulated in aged mice under hypertrophic stimulation, including pressure overload. Overexpression of Nox4 in the heart increased superoxide production and induced cardiac dysfunction accompanied by enhanced fibrosis and apoptosis. These authors also confirmed that Nox4 was localized primarily at mitochondria. 

 Surprisingly, Zhang et al. [21] found that Nox4 facilitated cardiac adaptation to chronic stress. In contrast to the other NADPH oxidase isoforms, Nox4 stimulation in cardiomyocytes led to the protection against pressure overload-induced adverse cardiac remodeling. Authors explain these surprising results by the Nox4-induced preservation of myocardial capillary density during pressure overload through the activation of hypoxia inducible factor 1 (Hif1) and the release of vascular endothelial growth factor (VEGF). They also found that the Nox4 location in cardiomyocytes was in perinuclear endoplasmic reticulum.

 Obviously these findings contradict those obtained by Kuroda et al. [19]. In both works Nox4-deleted mice were used but both groups of authors obtained contradictory data concerning the effects of Nox4 on oxidative stress in cardiac myocytes and Nox4 localization. Origin of that difference is unknown but it should be stressed that the data in [21] contradict a majority of findings concerning NADPH oxidase effects in cardiomyocytes and other cells.

### 2.3. NADPH Oxidase Nox1

Zhang et al. [22] suggested that Nox1 was a major NADPH oxidase isoform responsible for extracellular superoxide production in coronary arterial myocytes (CAMs).

## 3. ROS Production by Xanthine Oxidase in Heart and Cardiovascular Diseases

Principal reaction catalyzed by xanthine oxidase (XO) is the oxidation of xanthine into uric acid:

(2) $$\begin{array}{c} \text{XO}+{\text{H}}_{\text{2}}\text{O}+{\text{O}}_{\text{2}}\to \text{uric}\;\text{acid}+{\text{H}}_{\text{2}}{\text{O}}_{\text{2}} \end{array}$$

This catalytic process is accompanied by production of superoxide:

(3) $$\begin{array}{c} \text{XO}+{\text{O}}_{\text{2}}\to {\text{XO}}^{-1}+{{\text{O}}_{\text{2}}}^{•-} \end{array}$$

In contrast to the role of NADPH oxidase in heart diseases which was now ascertained with good certainty, the participation of xanthine oxidase in ROS-dependent cardiac injury caused earlier many doubts. Xanthine oxidase (XO) and xanthine dehydrogenase (XDH) are the oxidized and reduced forms of xanthine oxidoreductase (XOR). XO was considered to be a major producer of superoxide and hydrogen peroxide; its mechanism has been now well established [16, pages 701–703]. Paradoxically, McCord et al. [23] and Chambers et al. [24] suggested that xanthine oxidase was a main ROS producer in heart as early as in 1985 just about 5 years after epochal discovery of superoxide generation by xanthine oxidase [25]. Although this suggestion has been initially widely accepted, subsequent studies discovered a very low XO activity in animal and human hearts [26, 27]. 

 However, it is now generally agreed that there is a significant increase in cellular XO level and activity in the cardiovascular system under pathological conditions even though these changes may not be easily detected under physiological conditions. For example Thompson-Gorman and Zweier measured xanthine oxidase-mediated free radical generation in isolated rat heart [28]. They found that xanthine oxidase was an important factor of oxidative damage occurred upon the reperfusion of ischemic rat heart. It has been also shown that the burst of XO-catalyzed ROS production upon reperfusion was triggered by the enhancement of substrate concentration (xanthine and hypoxanthine) due to the degradation of ATP during ischemia [29]. Ashraf and Samra also suggested that XO activity increased during ischemia and intensified after reperfusion [30]. They found that XO was localized in interstitial cells, coronary vessel endothelium, and smooth muscle cells. De Jong et al. [31] showed that ROS production by xanthine oxidoreductase (XOR) increased in failing heart but not in hypertrophic heart. 

 Similarly to NADPH oxidases xanthine oxidase stimulated many ROS-dependent heart disorders. Landmesser et al. [32] showed that the increased XO activity and diminished extracellular superoxide dismutase (ecSOD) activity impaired endothelium-mediated vasodilation (FDD) in patients with chronic heart failure (CHF). In subsequent work Landmesser et al. [33] determined the XO protein levels and XO-dependent superoxide production in Ang II-induced endothelial cells from patients with coronary disease. They suggested that Ang II promoted superoxide overproduction by redox-sensitive XO activation.

 Duncan et al. [34] found that xanthine oxidase activity was elevated in a mouse model of dilated cardiomyopathy. Chronic inhibition of xanthine oxidase by allopurinol suppressed the progression of heart failure in dilated cardiomyopathy. Baldus et al. [35] found that ROS produced by xanthine oxidase impaired coronary NO bioavailability in patients with coronary artery disease (CAD). Minhas et al. [36] showed that the spontaneously hypertensive/heart failure (SHHF) rats exhibited the enhanced mRNA expression and activity of xanthine oxidoreductase (XOR), but XOR inhibition caused reverse remodeling in SHHF rats with dilated cardiomyopathy. Zhang et al. [37] demonstrated that myocardial ischemia/reperfusion (I/R) enhanced the expression of tumor necrosis factor (TNF-*α*) and induced the activation of xanthine oxidase and superoxide generation leading to coronary endothelial dysfunction in a murine model. Yamamoto et al. [38] showed that xanthine oxidoreductase and NADPH oxidase enhanced cardiac superoxide formation in Dahl salt-sensitive hypertensive rats with diastolic heart failure. Gonzalez et al. [39] demonstrated another toxic effect of superoxide generated by XO in spontaneously hypertensive heart failure rats with dilated cardiomyopathy. It was found that superoxide impaired S-nitrosylation of the ryanodine receptor (RyR) is responsible for calcium leak in sarcoplasmic reticulum (SR) of skeletal muscle.

## 4. ROS Production by Mitochondria in Heart and Cardiovascular Diseases

Mitochondria are an essential ROS producer in heart and vascular diseases although its significance as a ROS source comparing to NADPH oxidases and xanthine oxidase remains a subject for discussion. It is now well established that superoxide is generated by mitochondria due to electron leak from the two electron carriers of respiratory chain; these sources of superoxide are Complex I and Complex III [40]. Many authors confirmed importance of mitochondria-dependent ROS overproduction in heart damage. Thus Ide et al. [41] showed that mitochondrial Complex I was a potential ROS source in the failing myocardium in the canine hearts. Chen et al. [42] found that ischemia enhanced ROS production from the isolated rat heart mitochondria. Carpi et al. [43] demonstrated the crucial role of mitochondrial ROS formation in ischemia/reperfusion injury by the ablation of p66(Shc) protein in the mouse hearts. Chen et al. [44] showed that an increase in superoxide production (supposedly by mitochondria) was responsible for coronary endothelial dysfunction and decreased coronary blood flow (CBF) in congestive heart failure (CHF) in dogs. 

 Redout et al. [45] have studied ROS generation in the progression of ventricular hypertrophy to congestive heart failure. They found that both NADPH oxidase and mitochondria were the sources of ventricular ROS production in a rat model of right-ventricular (RV) heart failure induced by pulmonary arterial hypertension (PAH). Surprisingly, the enhanced activity of mitochondrial Complex II (and not Complexes I and III, major mitochondrial ROS producers) was particularly important for ventricular ROS production in heart failure. In contrast Mariappan et al. [46] showed that TNF-*α*-induced mitochondrial superoxide production impaired respiratory complex I activity and led to mitochondrial damage in the left ventricle (LV) in rats. Mitochondrial depolarization and enhanced ROS production mediated by lipoxygenase and arachidonic acid were probably responsible for arrhythmias following ischemia-reperfusion injury [47]. However, mitochondrial superoxide production in damaged heart might also induce the damage of mitochondrial electron transfer chain. Thus Chen et al. [48] found that superoxide suppressed the electron transfer activity of Complex II in postischemic myocardium in the rats subjected to coronary ligation followed by reperfusion. The aforementioned findings suggest that ROS overproduction by mitochondria might be an additional origin of heart disorders.

## 5. ROS and RNS Production by Nitric Oxide Synthases in Heart and Cardiovascular Diseases

Nitric oxide synthases (NOS) catalyze conversion of L-arginine to L-citrulline and nitric oxide but under uncoupling conditions these enzymes also produce superoxide:

(4) $$\begin{array}{cc} & \text{NOS}+\text{L-arginine}+{\text{O}}_{\text{2}}+\text{NADPH}\\ & \;\;\to \text{NO}+\text{citrulline}+{\text{NADP}}^{+}\\ & \text{NOS}\left(\text{Fe}\left(\text{II}\right)\text{heme}\right)+{\text{O}}_{\text{2}}\\ & \;\;\to \text{NOS}\left(\text{Fe}\left(\text{III}\right)\text{heme}\right)+{{\text{O}}_{\text{2}}}^{•-} \end{array}$$

Two NOS isoforms neuronal NOS (nNOS, NOS1) and endothelial NOS (eNOS, NOS3) are constitutively expressed in cardiomyocytes while inducible NOS (iNOS, NOS2) is absent in the healthy heart but its expression might be stimulated by prooxidants [49]. Thus Lijun et al. [50] showed that hypertrophied myocytes exhibited the elevated level of iNOS. However, Chen et al. [51] found the expression of all genes regulated NO synthases was reduced in the hearts of patients with coronary heart disease (CHD). 

 As NOSs are able to produce both RNS and ROS, the effects of these enzymes on cardiovascular system can be very complicated—they can enhance or diminish heart damage. Nitric oxide is the endothelium-derived relaxing factor (EDRF); therefore its function must be mainly favorable at the heart. However, the diffusion-controlled reaction of nitric oxide with superoxide produces the highly reactive peroxynitrite, a really harmful agent.

 Khadour et al. [52] have studied the formation of nitric oxide, superoxide, and peroxynitrite in the hearts from lipopolysaccharide- (LPS-) treated rats. They found an increase in the levels of all these reactive species in dysfunctional hearts from endotoxemic rats. Takimoto et al. [53] showed that pressure overload triggered the uncoupling of endothelial nitric oxide synthase NOS3 and enhanced mitochondrial superoxide production that induced dilatory remodeling and cardiac dysfunction in mice. Liu et al. [54] showed that the loss of inducible nitric oxide synthase (iNOS) in knockout mice (iNOS−/−) attenuated cardiac remodeling and dysfunction and improved cardiac reserve postmyocardial infarction (MI) probably due to a decrease in peroxynitrite formation. 

## 6. Interaction between ROS/RNS Produced Enzymes

Regulation of superoxide/nitric oxide balance is important for the prevention of heart damage. This balance is partly achieved by the interaction of principal enzymatic ROS producers during pathological changes in heart. Thus Saavedra et al. [55] showed that NO synthases and xanthine oxidase participated in the regulation of myocardial mechanical efficiency and that the upregulation of XO relative to NOS contributed to mechanoenergetic uncoupling in dogs with pacing-induced heart failure. Khan et al. [56] demonstrated that NOS1, as opposed to NOS3, directly interacted with xanthine oxidase in sarcoplasmic reticulum of cardiac myocytes in mice regulating cardiac excitation-contraction coupling. Therefore NOS1 is not only responsible for the regulation of calcium cycle in sarcoplasmic reticulum but also exhibits antioxidant activity through XO inhibition. 

 Saraiva et al. [57] suggested that the disruption of leptin (an adipose derived hormone) in the heart may contribute to obesity-related cardiac disease such as cardiac hypertrophy and enhanced cardiac apoptosis. They showed that leptin deficiency in mice was linked to decreased cardiac expression of NOS1 and NO production, with a concomitant increase in XOR activity and oxidative stress. Thus leptin is apparently able to change the NOS1/XO balance. Suematsu et al. [58] found that low sodium diet can induce the activation of rennin-angiotensin system, enhance superoxide generation by NADPH oxidase, and diminish NO bioavailability in the hearts of mongrel dogs. They suggested that low sodium might be responsible for increased mortality in patients.

## 7. ROS Signaling in Preconditioning

A brief episode of myocardial ischemia makes the heart remarkably resistant to a subsequent ischemia, the phenomenon named ischemic preconditioning. It has now been shown that ROS and RNS signaling play an important role in ischemic preconditioning and cardioprotection. For example it was found that 30 min of ischemia triggered by acetylcholine and an opioid receptor in isolated rabbit hearts stimulated preconditioning which included the activation of ROS- and RNS-dependent cascade of the epidermal growth factor (EGF) receptor, phosphatidylinositol 3-kinase (PI3-K), protein kinase B (Akt), nitric oxide synthase (NOS), and ROS-dependent opening of mitochondrial (mito)K(ATP) channels [59]. This cascade might also include extracellular signal-regulated kinase (ERK) located between Akt and nitric oxide synthase. 

 Kimura et al. [60] found that both mitochondria and NADPH oxidase mediated the preconditioning effects of Ang II in rat cardiac I/R injury in vivo through the enhanced cardiac mitochondria-derived ROS-initiated enzymatic cascade of NADPH oxidase/c-Jun amino-terminal kinase JNK and p38 MAPK protein kinases. Duda et al. [61] proposed that in guinea pig hearts ischemic preconditioning (IPC) raised endothelial protection by preventing postischemic endothelin-induced superoxide generation and the opening of mitochondrial ATP-dependent potassium channel (mK(ATP)). Superoxide was produced by NADPH oxidase and xanthine oxidase. Yue et al. [62] found that preconditioning in rat hearts can be stimulated by menadione-dependent superoxide production. Ischemic preconditioning reduced myocardial infarction by a mechanism that involved opening of mitochondrial ATP-dependent potassium channels (mK(ATP)), ROS formation, and possibly the activation of p38 mitogen-activated protein kinase (p38 MAPK).

 It is usually proposed that both K(ATP) channel opening and ischemic preconditioning protect the ischemic heart by acting at K(ATP) channels in the inner mitochondrial membrane. However Brennan et al. [63] found that in the isolated rat heart partial mitochondrial uncoupling induced by low-dose of the protonophore and uncoupler of mitochondrial oxidative phosphorylation FCCP significantly improved ROS-dependent postischemic functional recovery. This cardioprotection was not mediated by the depletion of cellular ATP or mitochondrial K(ATP) channel activation.

 Van-Cuong et al. [64] studied dynamic changes in nitric oxide produced by iNOS and eNOS and mitochondrial superoxide production during anoxic preconditioning (AP) in rat hearts. Under these conditions NO production played a pivotal role in scavenging of high levels of superoxide during post-anoxia/reoxygenation. These authors suggested that the continuous superoxide increase in preconditioning stimulated cell survival by the protection of cells from a sudden ROS shock at the onset of reoxygenation. Major signaling pathway in anoxic preconditioning was probably NO/cyclic guanosine monophosphate cGMP-protein kinase G (PKG)/ATP-sensitive potassium channel (KATP). Koneru et al. [65] demonstrated importance of the glucose transporter type 4 (GLUT-4) translocation and association in IP rat myocardium mediated by ROS signaling in Akt/eNOS/caveolin 3(Cav-3) enzymatic cascade. Juhaszova et al. [66] suggested that the mitochondrial permeability transition pore (mPTP) was a key end effector of ischemic/pharmacological pre- and postconditioning. Enhancement of ROS formation might induce mPTP-dependent resistance of cardiomyocytes to oxidant stress and infarct size reduction.

 Cohen et al. [67] have reviewed the mechanisms of enzymatic signaling pathways in ischemic preconditioning influenced by nitric oxide. They showed that the phosphorylation of nitric oxide synthase induced nitric oxide production, the subsequent activation of guanylyl cyclase, the production of cyclic guanosine monophosphate, the activation of protein kinase G, and opening of mitochondrial KATP channels. These processes were followed by ROS generation, which activated the critical kinase cascades of phospholipase C*δ*, PI3K, and ERK kinases. It is thought that these processes stimulate the inhibition of mitochondrial permeability transition pore formation and trigger the entrance into the preconditioned state. In recent work Vigneron et al. [68] showed that in the isolated-perfused mouse hearts preconditioning stimulated the downregulation of glycogen synthase kinase-3*β* (GSK-3*β*), the opening of mitoK(ATP), and ROS generation activating the target of rapamycin (mTOR) pathway and induced cardioprotection. These findings suggest that cardioprotection involved a prosurvival mTOR pathway.

## 8. Mechanisms of ROS and RNS Signaling in Heart and Cardiovascular Diseases

 Overproduction of ROS and deregulation of RNS production are important factors of the development of heart and cardiovascular diseases. Mechanisms of ROS and RNS generation by major producers, NADPH oxidases, xanthine oxidase, mitochondria, and nitric oxide synthases in these diseases as well as in preconditioning were discussed above and presented in Table 1(a). Now we will discuss the mechanisms of ROS and RNS signaling in heart pathological states.

### 8.1. ROS and RNS Signaling in the Processes Catalyzed by Protein Kinases B and C

Among various enzymes, protein kinases B and C and mitogen-activated protein kinases (MAPK) play a very important role in ROS and RNS-dependent enzymatic cascades responsible for heart damage. One of these kinases is the serine/threonine protein kinase B (Akt). It has already been noted that Akt participates in Nox2-initiated Ang II-dependent cardiomyocyte hypertrophy [11]. Recently Hingtgen et al. [86] demonstrated that an increase in cytoplasmic superoxide levels and Akt activation were responsible for the pressure overload-induced activation of nuclear factor NF-*κ*B and cardiomyocyte hypertrophy in mice. As it has been already noted, Akt also participates in preconditioning [59, 65]. There are examples of different signaling effects of Akt kinase. Chen et al. [69] found that NADPH oxidase-induced ROS activated Akt and ERK1/2 MAPK in cascade affected angiogenic growth factor expression and angiogenesis in mouse myocardium undergoing ischemia/reperfusion (I/R). Feng et al. [70] proposed that ROS levels affected NO-mediated coronary vasodilatation in mouse heart endothelial cells through NADPH oxidase-induced ROS/PI3-K/Akt/eNOS cascade. Akt signaling is also an important factor of gene-mediated processes (see below). 

 Participation of protein kinases PKC, PKC*δ*, and PKC*ε* in heart damaging processes has been also demonstrated [67, 71, 87]. Monti et al. [71] suggested that the activation of PKC kinases in coronary endothelial cells might influence the imbalance of eNOS/ROS system and endothelial dysfunction. It was found that selective inhibition of PKC*δ* or selective activation of PKC*ε* reduced oxidative damage in the heart following myocardial infarction. cGMP-dependent protein kinase (PKG) showed protective activity in the heart [88, 89].

### 8.2. ROS and RNS Signaling in Processes Catalyzed by Mitogen-Activated Protein Kinases MAPKS

Widder et al. [72] found that the activation of vascular p38 MAP kinase and its downstream target kinase MAPKAPK-2 in rats with heart failure was related to the elevated formation of superoxide and the reduction of NO bioavailability. They proposed that the activation of vascular p38 kinase in the heart failure caused the induction and activation of NADPH oxidase and superoxide overproduction. Gaitanaki et al. [73] showed that hyperthermia stimulated ROS-dependent activation of p38 and JNKs MAP kinases in the isolated perfused amphibian heart. Heusch et al. [74] demonstrated ROS formation, myofibrillar protein oxidation, and p38 kinase activation in failing rabbit heart. They found that p38 activation took place upstream rather than downstream of ROS formation which stimulated LV function through myofibrillar oxidation. Satoh et al. [75] showed that long-term treatment with17*β*-Estradiol (E2) improved congestive heart failure (CHF) in rats by antioxidative mechanism that involved thioredoxin (Trx) upregulation, the inhibition of Rac1(small GTPase-) mediated NADPH oxidase activity, and the apoptosis signal-regulating kinase 1(ASK-1)/JNK/p38-mediated apoptosis. Cai et al. [76] studied the effect of crocetin on cardiac hypertrophy. It was found that crocetin inhibited ROS-dependent MAPK [kinase kinase] (MEK)/ERK1/2 pathway responsible for hypertrophy in primary cultured cardiac myocytes and fibroblasts and in a one animal model of cardiac hypertrophy. Zhang et al. [90] showed that tumor necrosis factor TNF-*α* inhibited endothelium-dependent NO-mediated dilation of coronary arterioles from porcine heart by the ceramide-induced activation of JNK and subsequent production of superoxide by xanthine oxidase. 

 Younce and Kolattukudy [77] studied the role of MCP-1 (monocyte chemotactic protein-1) in the development of heart failure and apoptosis. They showed that MCP-1 caused death of cardiac myoblasts by inducing ROS formation and endoplasmic reticulum (ER) stress leading to autophagy *via* a novel zinc-finger protein, MCPIP (MCP-1-induced protein). MCPIP stimulated enzymatic cascade through the activation of MAP kinases JNK and p38. These findings suggested that MCPIP induced ROS/RNS production that stimulated ER stress, autophagy and apoptosis. Hikoso et al. [78] investigated the protective role of nuclear factor NF-*κ*B in cardiomyocytes in response to pressure overload. They demonstrated that I*κ*B kinase (IKK)-(NF-*κ*B) signaling cascade was protective in cardiomyocytes due to the attenuation of oxidative stress and JNK activation.

### 8.3. Other ROS-Dependent Enzymatic and Gene/Enzymatic Signaling Pathways in Heart Disorders

 It has been shown that tumor necrosis factor (TNF-*α*) is upregulated in a number of cardiomyopathies inducing adverse cardiac remodeling and dilation due to the degradation of the extracellular matrix by matrix metalloproteinases (MMPs). Awad et al. [79] found that recombinant TNF-*α* (rTNF) induced stronger superoxide production and increased expression of several MMPs in mouse neonatal cardiomyocytes comparing to cardiofibroblasts. Phosphatidylinositol 3-kinase (PI3K-*γ*) mediated TNF-dependent superoxide production and MMP expression. Lu et al. [80] have studied the expression of phosphodiesterase type 5 (PDE5) in left ventricular samples from patients with end-stage congestive heart failure (CHF) and normal donors and from mice after transverse aortic constriction-induced CHF. It was found that ROS increased PDE5 expression in cardiac myocytes and stimulated the CHF development in patients and in the mice. 

 Shan et al. [91] have studied myocardial ischemia/reperfusion (I/R) injury in diabetic hearts. They showed that the inhibition of Rac1signaling prevented NADPH oxidase activation and ROS overproduction resulting in the inhibition of calpain (calcium-activated neutral protease) activation. It was concluded that Rac1 activation increased I/R injury in diabetic hearts and was mediated by calpain activation. Zhao et al. [81] suggested that Ang II induced intracellular ROS production by NADPH oxidase and the activation of CaMKII (calmodulin kinase II) after depolarizations (EADs) and cardiac arrhythmias. Mechanisms of ROS signaling in harmful enzymatic heart processes are presented in Table 1. 

 It is known that several genes (p66Shc, sirtuin family, Klotho, and Foxo3a) regulate ROS formation in pathological states. Thus it has been shown that the suppression of *p66shc *gene stimulates stress resistance and prolongs lifespan in experimental animals. FOXO3a belonging to the O subclass of the forkhead family of transcription factors is considered to be a regulator of longevity and cancer. The effects of silent information regulator Sirtuin (human Sirt1 and Sirt3 and yeast Sir2) proteins depend on ROS levels and can stimulate or decrease longevity of experimental animals. Klotho gene regulates cell senescence and aging. Mechanisms of ROS regulation in such gene/enzymatic processes were recently discussed [92]. 

 These genes can also participate in ROS-dependent heart damage. Alcendor et al. [82] showed that Sirt1 was upregulated in response to low/moderate oxidative stress in adult mouse hearts causing the suppression age-dependent cardiac hypertrophy, apoptosis, and cardiac dysfunction. In contrast the high levels of Sirt1 enhanced these heart disorders. Sundaresan et al. [83] found that Sirt3 can block cardiac hypertrophic response in mice through the activation of Foxo-dependent antioxidative enzymes MnSOD and catalase and the suppression of ROS-mediated ERK and PI3K/Akt-dependent signaling pathways. 

 Guo et al. [84] showed that the p66Shc adapter protein participated in an *α*_1_-adrenergtic receptor (*α*_1_-AR) pathway together with protein kinases PKC*ε* and PKC*δ* and induced Akt-FOXO3a phosphorylation in rat cardiomyocytes. Sengupta et al. [85] showed that the regulation of transcription factors FoxO1 and FoxO3 by AMP-activated protein kinase (AMPK) and the activation of MnSOD and catalase were necessary for promotion of cardiomyocyte survival under conditions of oxidative stress (Table 1). The aforementioned findings demonstrate that the effects of genes in heart disorders might differ from those in the other pathological states. p66Shc is a negative regulator of cardiomyocyte hypertrophy and a major regulator of ROS production and cardiovascular oxidative stress responses. Thus p66Shc effects in heart diseases disagree with its effect on ROS stimulation in aging [92].

## 9. Antioxidants and Free Radical Scavengers as Suppressors of ROS-Induced Heart Disorders

For a long time antioxidants were considered important pharmacological agents for the treatment of heart diseases. It is impossible to discuss even a small part of works published on this problem (at present MEDLINE cites more 12400 works on *Heart Diseases and Antioxidants*), therefore we need to choose some principal studies. Antioxidants and free radical scavengers can suppress free radical-dependent heart disorders by direct reactions with reactive hydroxyl and peroxy free radicals or through the regulation of ROS signaling in gene- and enzyme-catalytic cascades. We will consider some important examples. 

 It has been shown that classical scavengers such as the grape-derived polyphenol resveratrol and synthetic phenol Probucol showed multifaceted cardioprotective activities [93, 94]. Another antioxidant recommended for the treatment of heart patients is L-arginine. Tripathi et al. [95] showed that oral administration of L-arginine to patients with angina and myocardial infarction might be protective due to an increase in plasma superoxide dismutase and total thiols. It should be noted that L-arginine is the substrate of NO synthases and therefore the enhancement of NO production can be another source of its protective action. 

 Superoxide is a one of two major signaling ROS, therefore the regulation of SOD activity can be important for the suppression of heart disorders. For example van Deel et al. [96] showed that the deficiency of EC-SOD (extracellular superoxide dismutase) gene enhanced oxidative stress in the left ventricular (LV), hypertrophy, and fibrosis in mice. These findings suggest that EC-SOD plays an important role in the heart protection against oxidative stress and infarction-induced ventricular hypertrophy. Gaitanaki et al. [97] found that ascorbic acid, SOD, or catalase inhibited ROS-induced activation of p38 MAPK in amphibian hearts. Ding et al. [98] showed that the transfer of adenoviral CuZnSOD (Ad CuZnSOD) gene to the carotid body increased CuZnSOD protein expression and reduced the baseline renal sympathetic nerve activity (RSNA) and the response of RSNA to hypoxia in rabbits with chronic heart failure. Recently Piantadosi et al. [99] showed that endogenous carbon monoxide (CO) generated by heme oxygenase-1 (HO-1) exhibited antioxidant effect by the stimulation of SOD-2 upregulation and mitochondrial production of hydrogen peroxide. It was suggested that the initiation of the HO-1/CO signaling through transcription factor Nrf2 and Akt kinase B stimulated the myocardial transcriptional program for mitochondrial biogenesis. Chen et al. [100] showed that omega-3 fatty acids prevented cardiac fibrosis and cardiac dysfunction by the activation of cyclic GMP/protein kinase G pathway in mouse cardiac fibroblasts. 

 Recent findings confirmed the protective effect of sildenafil (Viagra) against ischemia-reperfusion injury in the heart. It has been proposed that Sildenafil, a selective inhibitor of phosphodiesterase type 5 induced powerful protection against myocardial I/R injury through the activation of cGMP-dependent protein kinase G (PKG). Das et al. [101] suggested that the PKG-activated survival kinase ERK stimulated sildenafil-induced cardioprotection through the induction of eNOS/iNOS synthases. It was concluded that PKG-dependent ERK phosphorylation was the origin of eNOS/iNOS induction and cardioprotection by sildenafil. 

 Thus antioxidants of various classes might be considered to be of potential use for the prevention and maybe treatment of heart and cardiovascular diseases.

## 10. Discussion

The data discussed in this work show that practically all pathological disorders in heart and cardiovascular system are associated with damaging ROS signaling. These damaging processes can be initiated by ROS overproduced by various sources such as NADPH oxidases, mitochondria, xanthine oxidase, and NO synthases (Table 1), or normal physiological signaling processes might be converted into damaging ones by ROS-induced transformation of catalytic cascades. There are various factors which can be responsible for damaging ROS overproduction: aging, obesity, ischemia/reperfusion, hypertension, high-fat diet, low-molecular prooxidants, metal ions, and so forth [102–105]. 

 It is seen from Table 1 that ROS activate various enzyme/gene cascades in heart disorders. Protein kinases A-G and MAP kinases ERK, P38, and JNK apparently play an important role in these processes. However, the same enzymes are members of ROS-dependent normal physiological processes, therefore their transformation in pathological ones might be a consequence of ROS overproduction [40]. Indeed in many heart disorders (ischemia/reperfusion, changes of coronary vascular tone, myocardial infarction, LV dysfunction, cardiac hypertrophy, EAD, arrhythmias, or heart failure) prooxidant enzymes and proteins such as NADPH oxidases, Ang II, TNF-*α*, or monocyte chemotactic protein-1 (MCP-1) are responsible for ROS overproduction. ROS overproduction might be also initiated by cellular disorders for example initiated by hyperthermia (Table 1). 

 On the other hand ROS signaling in enzymatic/gene cascades might also decrease disorders in heart and cardiovascular system. As is seen from Table 1, PKC*δ* inhibition or PKC*ε* activation caused the suppression of oxidative stress and the reduction of myocardial infarction [71]. 17*β*-estradiol (E2) increased the expression of thioredoxin (Trx) and the suppression of NADPH oxidase/ASK-1/c-Jun/p38 cascade causing a decrease in apoptosis and the stimulation of CHF recovery [75]. Decrease in ROS signaling led to the reduction of cardiac hypertrophy and pressure overload in cardiomyocytes [76, 78]. 

 It is of interest that the participation of certain genes in signaling pathways frequently led to diminishing heart disorders. As is seen from Table 1, low ROS levels resulted in a decrease in hypertrophy, apoptosis, cardiac dysfunction, and cell senescence through moderate upregulation of Sirt1 while severe oxidative stress strongly upregulated Sirt1 and enhanced heart dysfunctions [82]. On the whole p66Shc and FOXO genes regulated ROS-dependent cascades diminishing hypertrophy and enhancing cell survival [84, 85]. ROS and RNS signaling under the conditions of moderate oxidative stress also leads to the suppression of heart disorders through preconditioning. 

 As ROS overproduction is one of the most important stimulators of heart disorders, it is logically to suggest that the application of antioxidants should be useful for fighting the heart and cardiovascular diseases. It has been shown above that the effects of antioxidants are very complicated and changed from direct interactions of antioxidants with reactive free radicals to the influence of enzymatic/gene pathways. For example protein kinases and the antioxidant/prooxidant genes can enhance or diminish ROS formation stimulating surviving and death of cardiomyocytes. 

 Another source of difficulty is dependence of the effects of antioxidants or prooxidants and various ROS inhibitors dependent on the levels of oxidative stress. As it was pointed out, low levels of ROS (low oxidative stress) might have prosurvival effects while high ROS levels (severe oxidative stress) will damage biomolecules. These effects can in turn be complicated by the survival effects of prooxidants-induced preconditioning and the induction of antioxidant enzymes (MnSOD and CuZnSOD). 

 Therefore a major question is how we might take into account all miscellaneous effects of antioxidants, prooxidants, and gene/enzymatic inhibitors for the selection of the right compounds and methods in the antioxidant (or prooxidant) treatment of heart diseases? Of course there is no simple answer. However, it is probably useful before beginning the treatment of patients to consider possible effects of the selected compound in major ROS and RNS signaling processes.

## Figures and Tables

**Table 1:**